# Considerations on diagnosis and surveillance measures of PTEN hamartoma tumor syndrome: clinical and genetic study in a series of Spanish patients

**DOI:** 10.1186/s13023-021-02079-7

**Published:** 2022-02-28

**Authors:** Laura Pena-Couso, María Ercibengoa, Fátima Mercadillo, David Gómez-Sánchez, Lucía Inglada-Pérez, María Santos, Javier Lanillos, David Gutiérrez-Abad, Almudena Hernández, Pablo Carbonell, Rocío Letón, Mercedes Robledo, Cristina Rodríguez-Antona, José Perea, Miguel Urioste, PHTS Working Group, Miguel Ángel Alonso, Raquel Andrés, Sara Arévalo, María del Mar Arias, Judith Balmaña, Elena Beristain, Ignacio Blanco, Mauro Boronat, Joan Brunet, María Victoria Cózar, Miguel del Campo, Arantza Díaz, Elisabeth Gabau, María Jesús Barcina, Margarita González, Miriam Guitart, Imma Hernán, Héctor Salvador Hernández, Susana Hernando, Carmen Lacambra, Adriana Lasa, Enrique Lastra, Gemma Llort, María del Rosario Marín, David Marrupe, Francisco Martínez, Víctor Martínez, Loreto Martorell, María Orera, Susana Pedrinaci, Pedro Pérez, Marta Pineda, Ana María Plasencia, Teresa Ramón y Cajal, Luis Robles, Diana Rodà, Nuria Rodríguez, Jordi Rosell, Raquel Sáez, Mónica Salvat, Antonio Sánchez, Alfredo Santana, José Luis Soto, Agustín Toll, Anna Tuneu, Carlos Vázquez

**Affiliations:** 1 Familial Cancer Clinical Unit, Spanish National Cancer Research Centre (CNIO), Madrid, Spain; 2 Respiratory Infection and Antimicrobial Resistance Group, Infectious Diseases Area, BioDonostia; Microbiology Department, Osakidetza Basque Health Service, Donostialdea Integrated Health Organization, San Sebastian, Spain; 3 Hereditary Cancer Laboratory, 12 de Octubre University Hospital, i+12 Research Institute, Madrid, Spain; 4 Clinical and Translational Lung Cancer Research Unit, i+12 Research Institute and Biomedical Research Networking Center in Oncology (CIBERONC), Madrid, Spain; 5 Biostatistics Unit, Statistics and Operational Research Department, Faculty of Medicine, Complutense University of Madrid, Madrid, Spain; 6 Hereditary Endocrine Cancer Group, Spanish National Cancer Research Centre (CNIO), Madrid, Spain; 7 Medical Oncology Service, University Hospital of Fuenlabrada, Fuenlabrada, Spain; 8 Dermatology Service, University Hospital of Fuenlabrada, Fuenlabrada, Spain; 9 Biochemistry and Clinical Genetics Centre, Virgen Arrixaca University Hospital, Murcia, Spain; 10 Rare Diseases Networking Biomedical Research Centre (CIBERER), Madrid, Spain; 11 Surgery Department, Fundación Jiménez Díaz University Hospital, Madrid, Spain; 12 Health Research Institute-Fundación Jiménez Díaz University Hospital, Madrid, Spain; 13 Virgen del Camino Hospital, Pamplona, Spain; 14 Lozano Blesa Hospital, Zaragoza, Spain; 15 Hospital of Donostia, Donostia, Spain; 16 Vall d’Hebrón Hospital, Barcelona, Spain; 17 Txagorritxu Hospital, Vitoria-Gasteiz, Spain; 18 Catalan Institute of Oncology, L’Hospitalet de Llobregat, Spain; 19 Hospital of Gran Canaria, Las Palmas de Gran Canaria, Spain; 20 Virgen de Valme Hospital, Sevilla, Spain; 21 Móstoles Hospital, Móstoles, Spain; 22 Parc Taulí Hospital, Sabadell, Spain; 23 Basurto Hospital, Bilbao, Spain; 24 Can Misses Hospital, Ibiza, Spain; 25 Terrassa Hospital, Terrassa, Spain; 26 Sant Joan de Déu Hospital, Esplugues de Llobregat, Spain; 27 Alcorcón Hospital, Alcorcón, Spain; 28 Severo Ochoa Hospital, Leganés, Spain; 29 Sant Pau Hospital, Barcelona, Spain; 30 Hospital of Burgos, Burgos, Spain; 31 Puerta del Mar Hospital, Cádiz, Spain; 32 Nuestra Señora de La Candelaria Hospital, Santa Cruz deTenerife, Spain; 33 La Paz Hospital, Madrid, Spain; 34 Gregorio Marañón Hospital, Madrid, Spain; 35 Virgen de Las Nieves Hospital, Granada, Spain; 36 San Carlos Hospital, Madrid, Spain; 37 Asturias Central Hospital, Oviedo, Spain; 38 12 de Octubre Hospital, Madrid, Spain; 39 Son Dureta Hospital, Palma, Spain; 40 Sant Joan de Reus, Tarragona, Spain; 41 Puerta de Hierro Hospital, Madrid, Spain; 42 General Hospital of Elche, Elche (Alicante), Spain; 43 Del Mar Hospital, Barcelona, Spain

**Keywords:** PTEN hamartoma tumor syndrome, Cowden syndrome, PTEN gene, NGS, Exome

## Abstract

**Background:**

The limited knowledge about the PTEN hamartoma tumor syndrome (PHTS) makes its diagnosis a challenging task. We aimed to define the clinical and genetic characteristics of this syndrome in the Spanish population and to identify new genes potentially associated with the disease.

**Results:**

We reviewed the clinical data collected through a specific questionnaire in a series of 145 Spanish patients with a phenotypic features compatible with PHTS and performed molecular characterization through several approaches including next generation sequencing and whole exome sequencing (WES). Macrocephaly, mucocutaneous lesions, gastrointestinal polyposis and obesity are prevalent phenotypic features in PHTS and help predict the presence of a *PTEN* germline variant in our population. We also find that PHTS patients are at risk to develop cancer in childhood or adolescence. Furthermore, we observe a high frequency of variants in exon 1 of *PTEN*, which are associated with renal cancer and overexpression of *KLLN* and *PTEN*. Moreover, WES revealed variants in genes like *NEDD4* that merit further research.

**Conclusions:**

This study expands previously reported findings in other PHTS population studies and makes new contributions regarding clinical and molecular aspects of PHTS, which are useful for translation to the clinic and for new research lines.

**Supplementary Information:**

The online version contains supplementary material available at 10.1186/s13023-021-02079-7.

## Background

The *PTEN* hamartoma tumor syndrome (PHTS; MIM 158350) encompasses several clinical entities with overlapping phenotypic characteristics, that are associated with germline pathogenic variants in the *PTEN* gene. Cowden syndrome (CS) and Bannayan-Riley-Ruvalcaba syndrome (BRRS) are the 2 principal entities of PHTS [1]. In addition to these, PHTS also includes some cases of Proteus-like syndromes, autism spectrum disorder (ASD) associated with macrocephaly, and VATER syndrome [2–5]. Macrocephaly, mucocutaneous lesions, Lhermitte-Duclos disease (LDD) and hamartomatous polyposis are generally considered characteristic features of PHTS. Heterozygous germline variants in the tumor suppressor gene *PTEN* cause PHTS. These variants are inherited in an autosomal dominant pattern and are found in up to 80–85% of CS patients, and in 60–65% of BRRS patients [2]. *PTEN* encodes a lipid and protein phosphatase that among other functions antagonizes the proliferative PI3K/AKT/mTOR pathway [6].

Besides numerous non-neoplastic multisystemic features, PHTS also entails increased risks for thyroid, breast, endometrial, colon, renal cell, and melanoma cancers [7]. These high cancer risks reinforce the importance of an early diagnosis that allows appropriate surveillance strategies. Reality, however, is quite the opposite: PHTS diagnosis usually takes a long time to be made, given the rareness of the disease, its considerable clinical heterogeneity, and the lack of awareness and the limited knowledge of this entity based on scarce studies. To improve this situation, several diagnostic criteria and recommendations have been proposed in the last decade [7–10]. However, these recommendations are based on a limited number of patients from only a few populations and there is still no consensus regarding their clinical and diagnostic utility [7, 9].

Furthermore, *PTEN* germline variants are not detected in a considerable number of patients who meet the clinical diagnostic criteria [2], reason why multiple efforts have been made to identify other genes involved in these syndromes. Variants in *SDH-B*, *SDH-D*, *PIK3CA*, *AKT1*, *TTN* and *SEC23B*, together with hypermethylation of the *KLLN* promoter, have been previously reported in certain CS, CS-like and BRRS patients [11–16].

Here, we describe the clinical and molecular aspects of 145 Spanish patients with a phenotype compatible with PHTS entities, to contribute to the knowledge of this rare disease by defining its characteristics in a new population and searching for other relevant genes.

## Results

### Spectrum of alterations in *PTEN*

Almost half of the patients (46% of the total series) had a germline alteration in the *PTEN* gene: 52 patients (36%) carried pathogenic point variants*,* 7 individuals (5%) had large deletions and 7 additional patients (5%) carried variants of unknown significance (VUS). The complete list of *PTEN* alterations is shown in Additional file 1: Tables S1-S3. No pathogenic variants nor VUS were found in the promoter of *PTEN*. We were able to trace the origin of the *PTEN* variants in 21 cases through genetic testing of their relatives. Thus, we confirmed the presence of 14 de novo (24% of the *PTEN* variant carriers) and 7 familial variant cases (12%).

**Fig. 1:**
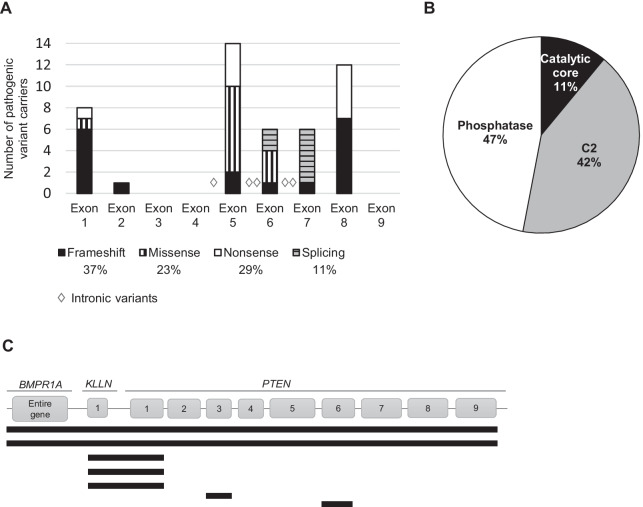
*PTEN* pathogenic variants found in our series. **A** *PTEN* point variants: numbers, distribution and types. **B** PTEN protein domains affected by the exonic variants. **C** Schematic representation of the gene regions affected by each of the 7 large deletions involving *PTEN*

We will refer to carriers of *PTEN* pathogenic variants and large deletions as *PTEN-*mut, excluding VUS carriers. The pathogenic variants appeared at higher frequencies in exons 5 to 8 of *PTEN*, but we also found a considerable number of patients carrying pathogenic variants in exon 1 (Fig. 1A). Several types of variants were observed (Fig. 1A) that affected the different PTEN protein domains, except the C-terminal end (residues 352 to 403) (Fig. 1B). Some pathogenic variants in *PTEN* appeared recurrently in our series: c.1003C > T, p.(Arg335*) (n = 5 unrelated patients); c.388C > T, p.(Arg130*) (n = 3); c.39_40del, p.(Arg14Glufs*29) (n = 2); c.406T > C, p.(Cys136Arg) (n = 2); and c.697C > T, p.(Arg233*) (n = 2).

Array comparative genomic hybridization (aCGH) allowed exhaustive characterization of 5 large deletions which also encompassed the proximal gene *KLLN*. Two of these rearrangements involved regions of 8 and 10 Mb, affecting other genes such as *BMPR1A* (Fig. 1C, Additional file 1: Table S3).

The remaining 79 probands (54%) were negative for pathogenic variants, large rearrangements or VUS in the *PTEN* gene. We will refer to these patients as *PTEN*-wt.

### Clinical characterization and criteria for *PTEN* study

The series of probands consisted mostly in adults (84%) but also included 25 patients (16%) under the age of 18 years old. 59% of the adults were women and 25% men, plus 12% young men and 4% young women. In the series of *PTEN*-mut+—excluding VUS—there were 32 women and 27 men. Mean age was 30 years in the mutation carriers (*PTEN*-mut+) and 46 in the PTEN-wt.

**Fig. 2:**
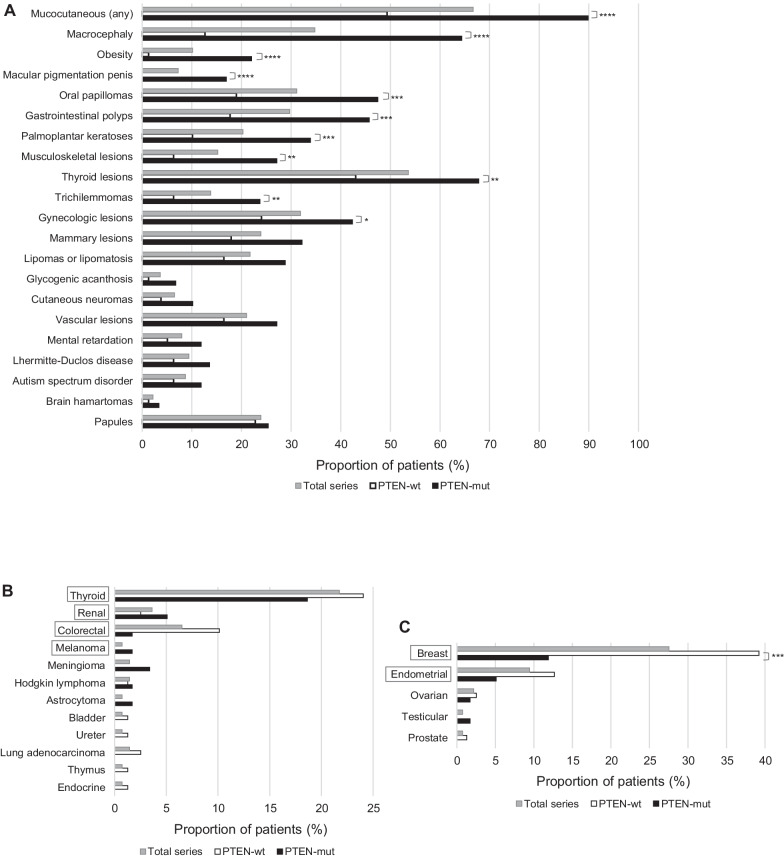
Proportion of individuals showing the indicated clinical manifestations in our series. **A** Occurrence of benign clinical features. **B** Occurrence of the different cancer types (not linked to sex). **C** Occurrence of sex-linked cancer types. PHTS-associated cancer types are boxed. Chi-square or Fisher test significance (*p < 0.05; **p < 0.01; ***p < 0.001; ****p < 0.0001) is shown for comparisons of absolute numbers of the *PTEN*-wt and *PTEN*-mut groups

Excluding the *PTEN* VUS carriers (described in Additional file 1: Table S2), we compared the phenotype between the *PTEN*-mut and the *PTEN*-wt groups, and found several clinical features that were significantly more common among *PTEN*-mut individuals, such as macrocephaly, mucocutaneous lesions and obesity (body mass index ≥ 30), suggesting their usefulness as criteria to identify *PTEN* pathogenic variant carriers (Fig. 2A). On the other hand, other classical clinical features of PHTS, such as LDD, ASD and vascular lesions, did not significantly discriminate between *PTEN*-mut and *PTEN*-wt individuals (Fig. 2A), suggesting they are poorer indicators of an individual carrying a *PTEN* pathogenic variant. This was supported by regression analyses (Additional file 1: Table S4).

The incidence of cancer was higher in the *PTEN*-wt compared to the *PTEN*-mut patients (58% (n = 46) versus 39% (n = 23); *p* = 0.0017). The 3 most frequent cancer types in the series (excluding the VUS carriers) were thyroid, colorectal and renal cancer, while breast, endometrial and ovarian cancers were the most frequent sex-linked cancers (Fig. 2B,C). Of the 69 cancer cases, 64 individuals had suffered some cancer within the spectrum of PHTS (PHTS-associated cancer): 20 *PTEN*-mut (31% of the PHTS-associated cancer patients) and 44 *PTEN*-wt (69%). Nevertheless, we also found other cancer types not so frequently associated with PHTS, like Hodgkin lymphoma, meningioma and astrocytoma in *PTEN*-mut patients (Fig. 2B, C).

We noticed that 18 patients of our series were referred for presenting only PHTS-associated cancers, with apparently no other feature of the disease. None of these individuals were carriers of *PTEN* pathogenic variants and they accounted for 23% of the *PTEN-*wt patients, suggesting that only the presentation of certain types of cancer might not be sufficient criterion to perform *PTEN* genetic testing. This is also supported by regression analyses, which shows a poor contribution of the presence of cancer alone to the probability of finding a *PTEN* pathogenic variant in the patient (Additional file 1: Table S5).

Interestingly, we found that a considerable proportion of our patients (14% of the *PTEN*-mut and only 3% of the *PTEN-*wt individuals; *p* = 0.013) developed cancer in childhood or adolescence (Table 1). Some of the cancer types we encountered are very rare in this age range, such as endometrial carcinoma or clear cell renal cell carcinoma. This suggests a risk to develop cancer at a very early age for carriers of germline pathogenic variants in *PTEN*.

**Table 1:** Cancer occurrence in individuals ≤ 18 years old from our series. Each case corresponds to a unique proband

Cancer type	Number of probands	Age of onset	*PTEN* germline status
Papillary thyroid cancer	1	16	p.(Lys6Argfs*4)
	1	16	p.(Arg335*)
Follicular thyroid cancer	1	14	p.(Arg335*)
Endometrial adenocarcinoma	1	15	p.(Cys136Arg)
Ovarian endodermal sinus tumor	1	6	p.(Cys136Arg)
Clear cell renal cell carcinoma	1	18	p.0?
Hodgkin lymphoma	1	18	p.(Gln17*)
Testicular mixed germ cell tumor	1	18	p.(Thr277Asnfs*21)
Hodgkin lymphoma	1	6	WT
Thyroid cancer (unspecified type)	1	14	WT

### Genotype–phenotype correlations

In order to search for genotype–phenotype correlations, we evaluated the clinical features as a function of the location of the variants along the *PTEN* sequence. We found several associations, most notably an association between renal cancer and pathogenic variants in *PTEN* exon 1 (*p* = 0.045; Additional file 1: Table S6).

### Search for other genetic factors

Given that not all patients with a clinical phenotype compatible with the PHTS entities are found to carry *PTEN* alterations, we searched for other genetic factors that could be involved as causal elements in these *PTEN*-wildtype patients. Considering the large variability of phenotypes found along the PHTS spectrum, we also searched for genes that could be involved as phenotype modifiers.

**Fig. 3:**
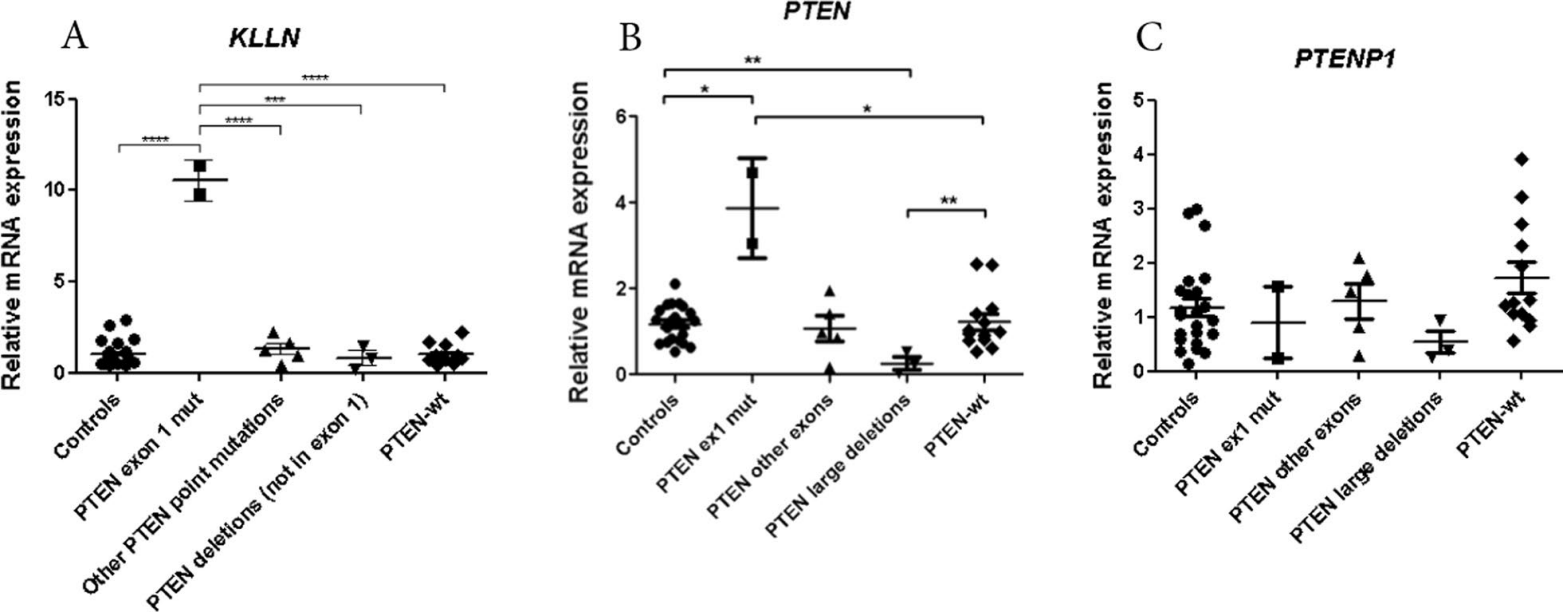
mRNA expression analyses. Relative mRNA expression of *KLLN* (**A**), *PTEN* (**B**) and *PTENP1* (**C**), determined by qPCR using *36B4* as a reference gene. Control individuals were compared to subgroups of PHTS patients according to the variant status of *PTEN*. Only significant differences are indicated (two tailed t-test; *p < 0.05; **p < 0.01; ***p < 0.001; **** p < 0.0001). Each dot represents the mean value of the expression of the gene under study for each patient (assessed in triplicate). The mean value of each group ± SEM is indicated

We first studied the mRNA expression of *KLLN* in our patient series. Strikingly, we found that the expression of *KLLN* was similar in patients and controls, except for patients carrying *PTEN* variants in exon 1, who showed overexpression of *KLLN* (Fig. 3A). These individuals also showed an unexpected high expression of *PTEN* (Fig. 3B), and we could rule out an upregulation of the pseudogene *PTENP1* (Fig. 3C). Therefore, only variants in exon 1 of *PTEN* seem to alter the expression levels of *PTEN* and its neighbor gene *KLLN*, although it is unclear how this contributes to PHTS etiology or pathogenesis. The only distinctive clinical feature of the patients with *KLLN* and *PTEN* overexpression was an increased presence of renal cancer, as noted above.

We also searched for alterations in mTOR pathway genes that could be involved either as phenotype modifiers in the *PTEN*-mut patients or as causal factors in the *PTEN*-wt patients. For this purpose, we screened almost our entire series (127 patients, including *PTEN*-mut and *PTEN*-wt) by using a multigene panel (see the full list of genes in Additional file 1: Table S7). None of the variants found (Additional file 1: Table S8) has been described to trigger the mTOR pathway and they were classified as VUS until additional experiments are performed. Moreover, the number of variants in mTOR pathway genes was similar in the groups of *PTEN*-mut and *PTEN*-wt patients, and we did not find any clear correlation between these variants and a specific phenotype in the carriers. The gene panel also targeted genes associated with other cancer predisposition syndromes (such as *FLCN*) and we found some *PTEN*-wt patients with rare variants in these genes (Additional file 1: Table S8). This prompted us to consider a clinical reevaluation of these patients.

In order to expand the search for genes involved in PHTS phenotypes other than *PTEN* and mTOR pathway genes, we performed WES in 11 unrelated *PTEN*-wt patients selected for their clinical features (see Methods) and we found several potentially relevant germline variants (Additional file 1: Fig. S1). On the one hand, we found variants in known cancer genes (such as *MUTYH*, associated with colorectal cancer risk) that might explain a certain feature of the patient’s phenotype, but not the development of a syndrome that resembles PHTS. On the other hand, we found several candidate variants to be further assessed in following studies (e.g. in *RNF135*, *NEDD4* and *HERC1*).

Additionally, available tumor DNAs were studied using the mentioned gene panel and the somatic variants found were in agreement with each cancer type, including a splicing variant in *PTEN* in the lung adenocarcinoma sample, and *BRAF* p.(V600E) and *PIK3CA* p.(G1049R) in thyroid cancer samples (Additional file 1: Table S9).

## Discussion

Our study describes clinical and molecular findings in a series of 145 patients with clinical features of PHTS entities, the largest one studied in the Spanish population thus far [17]. In agreement with previous studies in other populations [7, 8, 10], the pathogenic variants found in our series were located along the *PTEN* sequence, with hotspots in exons 5 and 8. In contrast to those other studies, however, we found a relatively high number of individuals with pathogenic variants in exon 1 [7, 8, 10]. All variants in exon 1 differed from each other, ruling out a founder effect. Our sample size does not allow us to support the conclusion that this is a specific characteristic of Spanish PHTS patients. The study of more individuals in other populations and in ours is required to better address this hypothesis.

When analyzing large rearrangements, we found several cases in which the deletion also affected other genes, such as *KLLN*, a neighbor gene of *PTEN*, but also *BMPR1A*, a further upstream gene associated with juvenile polyposis syndrome (JPS; MIM 174,900) and colorectal cancer risk. Hence, the 2 patients with a deletion affecting both *PTEN* and *BMPR1A* showed clinical features of both PHTS and JPS. This suggests an additive effect of the two genes and a possibly increased risk of colorectal cancer in these individuals. This finding is not uncommon in PHTS patients, as it has been previously reported [18–20]. Therefore, we recommend to extend the study of large deletion carriers by using methods such as aCGH or gene panels that allow copy number variant identification, in order to identify other genes that might also be deleted and cause additional clinical risks for the patient.

Our series of patients comes from very different medical specialists and not in all cases we have an exhaustive description of their clinical picture. Understanding that the percentages of each clinical trait should be interpreted as a minimum estimate, we can draw some valuable conclusions by comparing those patients with and without mutation in the *PTEN* gene.

As expected, several clinical features such as macrocephaly and mucocutaneous lesions were significantly more frequent among the *PTEN* pathogenic variant carriers, suggesting their usefulness as clinical diagnostic criteria. Together with these features, another good indicator of the presence of a *PTEN* pathogenic variant was obesity, present in 22% of our *PTEN*-mut individuals. Obesity rates in the Spanish population were: men 15.1%, women 13.1%, boys 10.6%, girls 11.8% (Global Obesity Observatory, https://data.worldobesity.org/country/spain-199/). This feature was hardly noted in the other patient series described in the literature [7, 8, 10]. Obesity has a very heterogeneous origin, and high rates observed in our series could be related with some features of the syndrome (e.g. thyroid disorders), but the association of PTEN and obesity could also be related with the involvement of PTEN in the insulin pathway [21, 22]. Despite of the limitations of our sample size it is important to highlight this finding since obesity is a known risk factor for cancer, with a strong causal link for breast, uterine, colon and renal cancers [23], and we observed that half (6 out of 13) of the obese *PTEN*-mut patients suffered cancer with a median onset age of 22 years old (versus 33 for non-obese patients). We therefore suggest that attention should be given to obesity in the surveillance of PHTS patients. In fact, it might be advisable to consider PI3K inhibitors in the treatment of PHTS patients not only to reverse classical PHTS lesions (such as skin hamartomas) [24], but also to treat obesity [25, 26].

On the other hand, some characteristics classically associated with CS, such as LDD (OR = 3.78, *p* = 0.11) and the presence of cancer (OR = 0.40, *p* = 0.05) alone, were poor predictors of a *PTEN* pathogenic variant. In the present study, we noticed that many clinicians find the presence of 2 or more CS-associated cancers a sufficient diagnostic criterion to refer a patient for *PTEN* testing. However, we found that all patients with CS-associated cancers but without any other reported clinical feature of CS (such as macrocephaly or mucocutaneous lesions) were *PTEN*-wt, i.e., none of them carried an alteration in *PTEN*. Therefore, the presence of CS-associated cancers should not be criterion to refer a patient for *PTEN* testing, unless this is accompanied by other features of the disease.

Cancer has been usually described to develop during adulthood in *PTEN* pathogenic variant carriers [7, 8, 10], and only one study highlighted the risk of early cancer development, especially thyroid cancer [27]. In fact, the guidelines of the U.S. National Comprehensive Cancer Network already suggest a yearly thyroid ultrasound for PHTS patients under 18 [28]. Based on our findings, 8 patients developed cancer under 18 among 59 patients PTEN-mut+, (Table 1), if a cancer is diagnosed at young age, it should be consider also genetic testing for *PTEN* variants. A large prospective study in young PHTS patients would be useful to establish the appropriate age at which screening should begin for each cancer type in these individuals.

We explored if the different clinical diagnostic criteria proposed in the literature [7, 9] could retrospectively identify the patients from our series (Additional file 1: Fig. S2). The International Cowden Consortium (ICC) and the Cleveland Clinic (CC) score had a good performance by identifying around 90% of our *PTEN*-mut patients (as expected, given the similarity of our criteria to these), together with 40% of the *PTEN*-wt patients. By contrast, when using the revised criteria proposed by Pilarski et al*.* [9] we failed to identify a considerable amount of the patients carrying *PTEN* pathogenic variants (more than 50% of these individuals).

To date, there are no strong genotype–phenotype correlations in PHTS, mainly due to the small sample sizes of the studies. Bearing in mind the limitations due to the size of our cohort, we did find some significant associations between the location of the *PTEN* pathogenic variant and the phenotype. Interestingly, we observed an association between renal cancer and *PTEN* exon 1 variants (*p* = 0.045). As *KLLN* was described as a possible phenotype modifier [15], we explored its role in our PHTS patients and found an overexpression of *KLLN* that correlated with an overexpression of *PTEN* (which may indicate a co-regulation of these genes due to their shared bidirectional promoter). However, no allelic–specific expression studies have been done, so we can´t determine if the upregulation is limited to the mutated allele. In consequence, the mechanism underlying this observation and its hypothetical association with an increase in risk for renal cancer in the individuals that harbored variants in *PTEN* exon 1, is currently unclear.

Since a considerable proportion of CS, CS-like, BRRS and ASD-macrocephaly patients (from 20 to 90%, depending on the clinical entity) do not carry a *PTEN* germline pathogenic variant [2–5], it is relevant to continue searching for new genetic factors involved in PHTS development, to improve counseling, risk assessment and therapeutic measures for each patient. Even though we did not find any clear candidate, several variants in known cancer predisposition genes (such as *FLCN*, *MUTYH* and *BAP1*) were observed, which made us reconsider the clinical diagnosis of these patients. As an example, one patient harbored a probably pathogenic variant in *FLCN*, a gene that is associated with the Birt-Hogg-Dubé (BHD) syndrome which includes cutaneous lesions that can resemble CS lesions. This example suggests the need for performing a differential diagnosis considering syndromes that have overlapping clinical features with the PHTS entities. We also performed WES and found some interesting variants that could account for etiological factors in *PTEN*-wt patients: variants in *RNF135*, associated with overgrowth, macrocephaly and facial dysmorphism [29]; variants in *UBN2*, associated with autism [30]; and variants in *NEDD4* and *HERC1*, which encode two ubiquitin ligases involved in PTEN and TSC2 degradation, respectively [31, 32]. We did not find candidate variants in *SDH-B*, *SDH-D*, *PIK3CA*, *AKT1*, *TTN* or *SEC23B*; these genes were suggested in the literature to be involved in CS and BRRS [11–14].

Several LDD patients do not present *PTEN* alterations and recently *EGFR* was proposed as a novel candidate for LDD susceptibility [33, 34]. Thus, we also sought for other LDD genes through WES in 4 *PTEN*-wt patients with this cerebellar tumor. We did not find any relevant alterations in *EGFR*, but of note, we found an as yet unreported variant in *FGFR1*, which encodes a receptor involved in PI3K signaling [35, 36].

ASD has a complex etiology, with at least 1,000 susceptibility genes reported [30] and variants in *PTEN* account for a relevant amount of individuals with ASD and macrocephaly [37]. Five *PTEN*-wt patients of our series showed this phenotype. Through WES in one of these individuals, we found a missense variant in the *ATR* gene (malfunction of its protein can impair fragile site stability, which can be a risk for autism), a stop gain variant in *UBN2* and two different variants in *EP400*; variants in the latter two genes have been suggested to be associated with autism [30].

We found several variants in other genes besides *PTEN* that could account for a subset of the patients who tested negative for alterations in *PTEN*, but their importance remains to be determined before a translation to the clinical setting can be considered. Moreover, we did not find a common gene altered in several patients, similar to the results of other authors [38, 39], and only certain gene variants could explain specific individual cases. Therefore, it is possible that *PTEN* is the only high susceptibility gene of CS, CS-like, BRRS or ASD-macrocephaly, and other yet to be discovered factors might explain the disease in individuals with no variants in *PTEN*. Other approaches such as RNA-sequencing, genome sequencing or methylation assays might shed light on this issue.

The fact that the somatic variants found in our work in the cancer tissues were to be expected for each cancer type could point towards a similar evolution pattern of tumors in PHTS and their sporadic counterparts, although we were only able to study a small sample set.

## Conclusions

Our findings suggest that to improve diagnosis, focus should be put on macrocephaly, mucocutaneous lesions, obesity and gastrointestinal polyposis when performing the clinical evaluation, as these were features that best suggested the presence of a *PTEN* pathogenic variant. Once the *PTEN* germline status of the patient is known, it is relevant to perform a differential diagnosis in case no pathogenic variants were found. In this last scenario or when finding large rearrangements, it is important to expand the search to other genes that might be altered causing additional or unexpected clinical risks. Finally, regarding the management and follow-up recommendations for PHTS, we suggest regularly monitoring weight and considering cancer screenings at an earlier age in young individuals. Prospective studies of PHTS patients will aid in the determination of their clinical risks.

## Materials and methods

### Patients and clinical evaluation

One hundred and forty-five probands (unrelated individuals from unique families) meeting relaxed clinical criteria from the International Cowden Consortium (ICC) [7], including patients who a) met the pathognomonic criteria, b) met 1 major criterion and 2 minor criteria, and c) suffered any 2 of the following cancer types within the PHTS spectrum: breast, thyroid or endometrial cancer, were included in our series. All patients were Caucasians of Spanish origin (age range from 1 to 76 years old; 84% adults and 16% ≤ 18 years old). 26 patients were seen in the consultancy of our group at the University Hospital of Fuenlabrada (UHF, Madrid, Spain). The remainder 119 patients were referred to our laboratory through collaboration with medical specialists of 35 different hospitals from Spain (PHTS Working Group). Biological samples, clinical information and signed informed consent were referred to our laboratory at the Familial Cancer Clinical Unit (CNIO, Madrid, Spain). Written formal consent was obtained from the parents or guardians of individuals under 18 years old. The patients’ phenotype information was collected through a clinical questionnaire specifically designed for this project (Additional file 4: Methods S1). We sent 119 questionnaires to medical professionals from 9 different specialties. 51 questionnaires were returned to us completed. The project has the approval of the Ethics Committee of the UHF (approval number: 20/28).

### *PTEN* genetic analyses

DNA was extracted from samples of peripheral blood leukocytes of each proband. The presence of germline variants was evaluated in all 9 exons of *PTEN* (together with the intron–exon boundaries) by PCR and Sanger sequencing. Variants were named in relation to NM_000314.4. Positive results were confirmed in a second blood sample using the MyTaqBlood PCR (Bioline) reagent. Large rearrangement analysis was performed through multiplex ligation-dependent probe amplification (MLPA) with SALSA P225D1 (MRC Holland). The SurePrint G3 Unrestricted CGH 4x180K microarray (Agilent) was used for cases with large deletions involving the 5’ end of *PTEN* to interrogate the extent of the deletion and the location of the breakpoints. The *PTEN* promoter was analyzed in 31 patients in whom no potentially relevant changes in the *PTEN* gene were detected. Primers are mentioned in Additional file 5: Methods S2.

### mRNA expression analyses

RNA was extracted from peripheral blood leukocytes with TRIzol reagent (Thermo Fisher). Concentration and integrity of the RNA were checked using NanoDrop (ND-1000 V3.7.1; Thermo Fisher). The High Capacity cDNA reverse transcriptase kit (Applied Biosystems) was used to synthesize cDNAs. cDNAs from unaffected donors were used as controls for expression levels in the qPCR reaction. Samples (n = 23) and controls (n = 23) were analyzed in triplicate. Primers for *PTEN*, *KLLN* and *PTENP1* are listed in Additional file 4: Methods S1. The GoTaq® qPCR Master Mix (Promega) was used for the reaction and the qPCR was performed in an ABI QuantStudio S6 Flex System (Applied Biosystems). *36B4* was used as the reference gene to calculate relative mRNA expression using the 2^−∆∆Ct^ method for qPCR analysis.

### Next generation sequencing (NGS) panel

A total of 131 DNA samples (127 from blood and 4 from paraffin-embedded tumor tissue samples) were included in a custom NGS panel from NimbleGen (Roche) to look for other possible genetic factors involved as phenotype modifiers or with a causal role. Variant filtering consisted of maintaining only the variants in canonical transcripts (APPRIS) with high or moderate functional effect—refer to the Variant Effect Predictor (VEP, Ensembl) calculated impacts, with moderate impact corresponding to coding non-synonymous variants (e.g. missense, in-frame indels) and high to loss-of-function variants (e.g. nonsense, those disrupting canonical splice sites, frameshift indels)—, a variant allele frequency (VAF) between 0.3 and 0.6 for heterozygotes and > 0.9 for homozygotes, and a minor allele frequency (MAF) < 0.1% (gnomAD). Somatic variants were called with Mutect2 (GATK4, ref) and variants were filtered out following the following criteria: “LOW” and “MODIFIER” categories according to VEP; variants present in the matched-paired blood sample (germline variants); variants with a VAF < 15% “ were excluded. Further details are described in the Additional file 4: Methods S1 and Additional file 1: Table S7.

### Whole exome sequencing (WES)

Eleven individuals with no pathogenic variants or variants of unknown significance (VUS) identified in *PTEN* (7 meeting Pilarski’s clinical diagnostic criteria [9], 3 with LDD, and 1 pediatric case with macrocephaly, autism and overgrowth) were selected for whole exome sequencing (WES). Germline genomic DNA samples from these individuals were quantified using Quant-iT PicoGreen dsDNA reagent (Thermo Fisher), their quality was checked using the NanoDrop spectrophotometer (ND-1000 V3.7.1; Thermo Fisher) and degradation was assessed by agarose gel electrophoresis. WES (12 Gb, 100X coverage) and primary bioinformatics analysis were performed at the Novogene Bioinformatics Institute (Beijing, China). Further details are described in the Additional file 4: Methods S1.

### Interpretation and validation of variants

Variants were considered deleterious if they were described as such in public databases (ClinVar, HGMD and LOVD), or when the specific study of cDNA sequence supported a deleterious consequence. The presence of variants was confirmed in a second sample using a different method: MyTaq Blood-PCR Kit (Bioline).

### Statistical analyses

Chi-square or Fisher’s exact tests were used to evaluate differences between our cohort and other previously published cohorts [7, 8, 10] using R. Logistic regression was used to evaluate associations and risks using SPSS. qPCR analyses (t-test, Mann–Whitney) were done using GraphPad Prism (GraphPad Software, Inc.). Bilateral *p* values < 0.05 were considered statistically significant.

## Supplementary Information

**Additional file 1: Fig. S1.** Clinical manifestations and selected gene variants for the 11 *PTEN*-wt patients studied through WES. Group A: patients who meet strict Pilarski* diagnostic criteria; group B: patients who developed LDD but do not meet strict Pilarski* diagnostic criteria; group C: pediatric case with macrocephaly together with neurological alterations and overgrowth. *Pilarski et al. 2013. **Fig. S2**. Proportion of individuals in each group of our series identified using the indicated diagnostic criteria from the literature. (See references in Additional file 3: Appendix S2). **Table S1.** *PTEN* pathogenic point variants found in our series. Variants are named in relation to NM_000314.4. **Table S2.** *PTEN* variants of unknown significance (VUS) found in our series. Variants are named in relation to NM_000314.4. **Table S3.** Deleted chromosomal regions found in our series through MLPA and aCGH. **Table S4.** Risk of carrying a *PTEN* germline pathogenic variant according to different clinical features, based on analysis of our patient series. Odds ratios obtained with logistic regression. **Table S5.** Risk of carrying a *PTEN* germline pathogenic variant when the patient presents only a certain clinical feature. Odds ratios obtained with logistic regression. **Table S6.** Genotype–phenotype associations found in our series. Chi-square or t-test significance is shown. **Table S7.** List of genes included in the multigene panel. **Table S8.** Germline VUS found in mTOR-related genes by NGS. **Table S9.** Germline *PTEN* status and NGS findings in the indicated tumor samples (each one from a different proband).

**Additional file 2: Appendix S1.** List of authors and affiliations of the PHTS working group.

**Additional file 3: Appendix S2.** References related to supporting information.

**Additional file 4: Methods S1.** Clinical questionnaire.

**Additional file 5: Methods S2.** Primers and further information regarding the NGS and WES.

## Data Availability

The dataset of current study are not publicly available but are available from the corresponding author on reasonable request.

## Abbreviations

aCGH: Array comparative genomic hybridization
ASD: Autism spectrum disorder
BHD: Birt-Hogg-Dubé
BRRS: Bannayan-Riley-Ruvalcaba syndrome
CC: Cleveland Clinic
CS: Cowden syndrome
HGMD: Human gene mutation database
ICC: International Cowden Consortium
LDD: Lhermitte-Duclos disease
JPS: Juvenile polyposis syndrome
LOVD: Leiden open variation database
MAF: Minor allele frequency
MLPA: Multiplex ligation-dependent probe amplification
PHTS: PTEN hamartoma tumor syndrome
NGS: Next generation sequencing
VAF: Variant allele frequency
VATER: Vertebral, anal, tracheal, esophageal and renal abnormalities
VUS: Variant of unknown significance
WES: Whole exome sequencing
