# Supplementary material for: Considerations on diagnosis and surveillance measures of PTEN hamartoma tumor syndrome: clinical and genetic study in a series of Spanish patients
Source: Orphanet J Rare Dis. 2022 Feb 28;17:85. doi: 10.1186/s13023-021-02079-7 (PMC8886852; doi:10.1186/s13023-021-02079-7)
Supplement: Supplementary file 1 — Additional file 1: Fig. S1. Clinical manifestations and selected gene variants for the 11 PTEN-wt patients studied through WES. Group A: patients who meet strict Pilarski* diagnostic criteria; group B: patients who developed LDD but do not meet strict Pilarski* diagnostic criteria; group C: pediatric case with macrocephaly together with neurological alterations and overgrowth. *Pilarski et al. 2013. Fig. S2. Proportion of individuals in each group of our series identified using the indicated diagnostic criteria from the literature. (See references in Additional file 3: Appendix S2). Table S1. PTEN pathogenic point variants found in our series. Variants are named in relation to NM_000314.4. Table S2. PTEN variants of unknown significance (VUS) found in our series. Variants are named in relation to NM_000314.4. Table S3. Deleted chromosomal regions found in our series through MLPA and aCGH. Table S4. Risk of carrying a PTEN germline pathogenic variant according to different clinical features, based on analysis of our patient series. Odds ratios obtained with logistic regression. Table S5. Risk of carrying a PTEN germline pathogenic variant when the patient presents only a certain clinical feature. Odds ratios obtained with logistic regression. Table S6. Genotype–phenotype associations found in our series. Chi-square or t-test significance is shown. Table S7. List of genes included in the multigene panel. Table S8. Germline VUS found in mTOR-related genes by NGS. Table S9. Germline PTEN status and NGS findings in the indicated tumor samples (each one from a different proband). [file 13023_2021_2079_MOESM1_ESM.pdf]

Supplementary figures

Figure S1

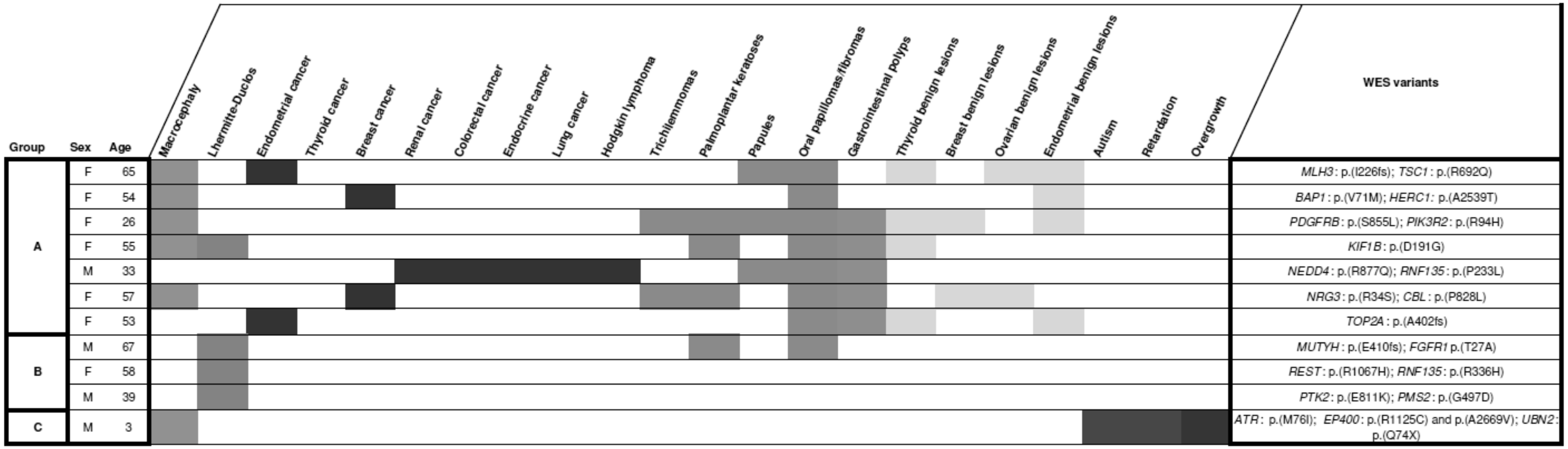

Figure S1. Clinical manifestations and selected gene variants for the 11 *PTEN*-wt patients studied through WES. Groups A: Patients who meet strict Pilarski[8] diagnostic criteria; group B: patients who developed LDD but do not meet strict Pilarski[8] diagnostic criteria; group C: pediatric case with macrocephaly together with neurological alterations and overgrowth.

Figure S2

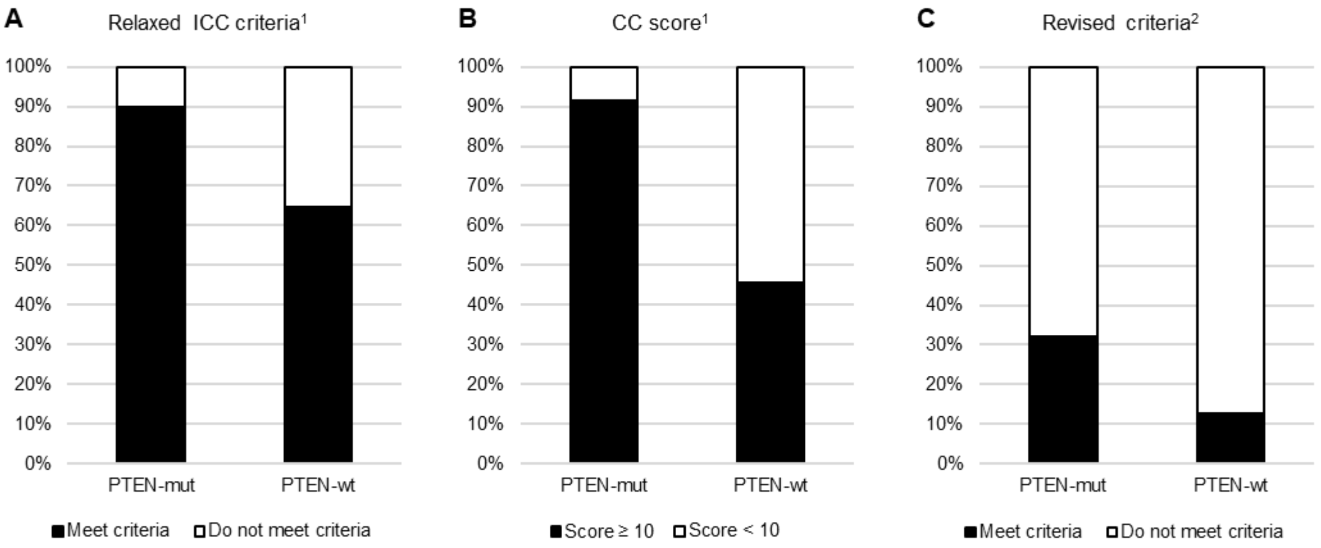

Figure S2. Proportion of individuals in each group of our series identified using the indicated diagnostic criteria from the literature (references are listed at the end of this document).

Supplementary tables

Table S1. *PTEN* pathogenic point variants found in our series.

Sample ID	DNA change	Protein change
803F	c.1_2delAT	p.0?
219F	c.17_18del	p.(Lys6Argfs*4)
186F	c.39_40del	p.(Arg14Glufs*29)
864F	c.39_40del	p.(Arg14Glufs*29)
700F	c.49C>T	p.(Gln17*)
427F	c.58_61dup	p.(Phe21Trpfs*24)
30F	c.68_69insA	p.(Asp24fs*20)
1041F	c.81_82insCT	p.(Ile28Leufs*27)
355F	c.254-1_257dup	
1166F	c.255_256insAA	p.(Ala86Lysfs*14)
683F	c.302T>C	p.(Ile101Thr)
517F	c.332G>A	p.(Trp111*)
1126F	c.334C/G	p.(Leu112Val)
249F	c.379G>A	p.(Gly127Arg)
55F	c.388C>T	p.(Arg130*)
614F	c.388C>T	p.(Arg130*)
653F	c.388C>T	p.(Arg130*)
757F	c.389G>A	p.(Arg130Gln)
532F	c.395G>T	p.(Gly132Val)
794F	c.405dup	p.(Cys136Metfs*44)
1157F	c.406T>C	p.(Cys136Arg)
1251F	c.406T>C	p.(Cys136Arg)
910F	c.407G>A	p.(Cys136Tyr)
836F	c.492+1delG	
582F	c.492+1G>C	
44F	c.493-1G>A	p.(Gly165fs*)

Sample ID	DNA change	Protein change
724F	c.493-1G>C	p.(Gly165Ilefs*9)
817F	c.493G>C	p.(Gly165Arg)
805F	c.512A>G	p.(Gln171Arg)
777F	c.542T>C	p.(Leu181Pro)
232F	c.622_623insT	p.(Gly208Valfs*)
189F	c.634+5G>A	
250F	c.635-1G>C	
1085F	c.640C>T	p.(Gln214*)
788F	c.655C>T	p.(Gln219*)
1020F	c.686C>G	p.(Ser229*)
1274F	c.68T>G	p.(Leu23*)
499F	c.697C>T	p.(Arg233*)
518F	c.697C>T	p.(Arg233*)
762F	c.723_724insTT	p.(Glu242Leufs*15)
578F	c.817_818del	p.(Phe273*)
659F	c.825del	p.(Val275*)
1089F	c.829dup	p.(Thr277Asnfs*21)
981F	c.955_958del	p.(Thr319*)
120C	c.984_987del	p.(Asn329Lysfs*)
705F	c.985_986delAA	p.(Asn329*)
1105F	c.1003C>T	p.(Arg335*)
262F	c.1003C>T	p.(Arg335*)
687F	c.1003C>T	p.(Arg335*)
750F	c.1003C>T	p.(Arg335*)
795F	c.1003C>T	p.(Arg335*)
621F	c.1007dup	p.(Tyr336*)

Table S2. *PTEN* variants of unknown significance (VUS) found in our series.

Patient ID	DNA change (ClinVar submission ID)	Protein change	Protein domain	Deleteriousness prediction (Condel)	Phenotype	Variant origin
789F	c.77C>T (SCV001245482)	p.(Thr26Ile)	Phosphatase	Probably damaging	Male 11 yo. Macrocephaly, obesity, macular pigmentation penis, bilateral gynecomastia, autism, mental retardation, musculoskeletal alterations.	*De novo*
707F	c.254-21G>C (SCV001245485)	-	-	-	Male 10 yo. Macrocephaly, autism, hyperpigmented spots.	Unknown. (Relatives were not tested).
1170F	c.284C>G	p.(Pro95Arg)	Phosphatase	Probably damaging	Male 39 yo. Macrocephaly, overweight, papules, palmoplantar keratoses, trichilemmomas, oral papillomatosis, lipomas, macular pigmentation penis, goiter, Hashimoto's thyroiditis, colonic and gastrointestinal polyps, cerebral hamartomas.	Unknown. (Relatives were not tested).
1201F	c.529T>A (SCV001245483)	p.(Tyr177Asn)	Phosphatase	Probably damaging	Male 5 yo. Macrocephaly, motor delay.	Familial. (Variant segregates in the family with the phenotype).
738F	c.781C>G (SCV001245484)	p.(Gln261Glu)	C2	Probably damaging	Male 34 yo. Macrocephaly, lipomas, macular pigmentation penis, colorectal polyps, general overgrowth.	Familial. (Variant segregates in the family with the phenotype).
1115F	c.829A>G	p.(Thr277Ala)	C2	Probably damaging	Male 9 yo. Macrocephaly, general developmental disorder, speech delay, general overgrowth.	*De novo*
1103F	c.929A>G	p.(Asp310Gly)	C2	Probably neutral	Male 29 yo. Macrocephaly, papules, palmoplantar keratoses, thyroid adenomas, papillary-follicular thyroid cancer, colorectal polyps (inflammatory, lymphoid and hamartomatous), testicular cancer.	Unknown. (Only a sister was tested; she was a non-carrier).

Table S3. Chromosomal regions deleted found in our series through MLPA and aCGH.

ID	Deleted region (MLPA)	Deleted region (aCGH)
594F	Entire *PTEN**	chr10:81,685,169-91,936,008 (10 Mb)
617F	*BMPR1A*, *KLLN* and entire *PTEN*	chr10:81,660,274-89,830,454 (8 Mb)
708F	*KLLN* and *PTEN* exon 1	chr10:89,625,664-89,640,157 (14 kb)
858F	*KLLN* and *PTEN* exon 1	chr10:89,625,664-89,640,157 (14 kb)
814F	*KLLN* and *PTEN* exon 1	chr10:89,653,535-89,653,594 (59 pb)
712F	*PTEN* exon 6	-
1054F	*PTEN* exon 3	-

*Study performed in 2006 with a probe kit that did not include other genes besides *PTEN;* the remaining DNA could not be used to repeat the study.

Table S4. Risk of carrying a *PTEN* germline mutation according to different clinical features, based on analysis of our patient series. Odds ratios obtained with logistic regression.

Clinical feature	OR	CI95	*P* value
**Macrocephaly**	**11.186**	4.88 - 25.67	**1.21E-08**
**Mucocutaneous lesions**	**7.619**	3.09 - 18.82	**1.07E-05**
**Gastrointestinal polyps**	**4.488**	2.08 - 9.7	**0.1E-03**
Lhermitte-Duclos disease	2.322	0.72 - 7.50	0.159
**Obesity**	**23.045**	2.91 - 182.14	**0.003**
Breast cancer	0.208	0.08 - 0.52	0.001
Thyroid cancer	0.724	0.31 - 1.67	0.447
Endometrial cancer	0.502	0.15 - 1.69	0.265
Renal cancer	2.062	0.33 - 12.76	0.436
Colorectal cancer	0.153	0.02 - 1.26	0.081
Family history	1.477	0.64 - 3.40	0.359
No. of cancers per patient	0.546	0.36 - 0.82	0.004
Cancer onset age	0.907	0.86 - 0.96	0.001

Table S5. Risk of carrying a *PTEN* germline mutation when the patient presents only a certain clinical feature (for example, a patient who has only LDD and no other clinical manifestation). Odds ratios obtained with logistic regression.

Clinical feature	OR	CI95	*P* value
Cancer	0.399	0.16 - 1.01	0.052
**Macrocephaly**	**11.385**	**4.29 - 30.21**	**1.04E-06**
**Mucocutaneous lesions**	**6.861**	**2.18 - 21.60**	**0.001**
**Gastrointestinal polyps**	**4.220**	**1.60 - 11.16**	**0.004**
Lhermitte-Duclos disease	3.776	0.74 - 19.29	0.110

Table S6. Genotype-phenotype associations found in our series. Chi-square or t-test significance is shown.

***PTEN* genotype**	**Phenotype (*p* value)**
Mutations in exon 1	↑Renal cancer (p = 0.045)<br>↑Palmoplantar keratosis (p = 0.01)<br>↑Papules (p = 0.02)
Mutations in exon 8	↑Musculoskeletal lesions (p = 0.03)
Large deletions	↓Polyposis (gastric: p = 0.047, colorectal: p = 0.017)<br>↓Neurological disorders (autism: p = 0.03, mental retardation p = 0.03).

S7 Table. List of genes included in the multigene panel.

Nr	Gene	Nr	Gene
1	*AKT1*	24	*FLCN*
2	*AKT2*	25	*TP53*
3	*AKT3*	26	*FH*
4	*DEPDC5*	27	*MET*
5	*MTOR*	28	*CDKN2A*
6	*PIK3C2B*	29	*AR*
7	*PIK3CA*	30	*KRAS*
8	*PIK3R1*	31	*BRAF*
9	*PTEN*	32	*NRAS*
10	*RICTOR*	33	*MEN1*
11	*RPTOR*	34	*DAXX*
12	*STK11*	35	*ATRX*
13	*TSC1*	36	*CUL3*
14	*TSC2*	37	*NF1*
15	*NF2*	38	*ARID1A*
16	*SETD2*	39	*CDKN1B*
17	*SMARCB1*	40	*EIF1AX*
18	*USP9X*	41	*NFE2L2*
19	*BAP1*	42	*STAG2*
20	*KDM5C*	43	*TERT*_region ATG (250 nt of the promoter)
21	*KDM6A*	44	*TFE3* fusion (ex2-ex6; incl. intr2-5; ENST00000315869.7)
22	*PBRM1*	45	*TFEB* fusion (1 kb intr2-ex3; ENST00000230323.8)
23	*VHL*	46	*TFEB* fusion (ex9, intr9, ex10; ENST00000230323.8)

Table S8. Germline VUS found in mTOR-related genes by NGS.

ID	Patient group	Variant	Gene	AF	Consequence	IMPACT	Ref gene	Protein position	Amino acids	Existing variation	gnomAD AF	Clin_sig
1105F	PTEN-mut	1:11273527G>A	*MTOR*	0.46	missense	MODERATE	ENSG00000198793	1072	P/S			
1251F	PTEN-mut	1:204418411C>T	*PIK3C2B*	0.49	missense	MODERATE	ENSG00000133056	458	R/Q	rs61755372	0.000658	
30F	PTEN-mut	16:2111938G>T	*TSC2*	0.50	missense	MODERATE	ENSG00000103197	396	D/Y	COSM6143684&COSM6143685		
497F	PTEN-mut	16:2122869T>A	*TSC2*	0.50	missense	MODERATE	ENSG00000103197	747	L/Q	CD010683		
499F	PTEN-mut	14:105246462G>T	*AKT1*	0.40	missense	MODERATE	ENSG00000142208	232	G/E			
499F	PTEN-mut	9:135771994G>C	*TSC1*	0.53	missense	MODERATE	ENSG00000165699	692	R/Q	rs199755731	0.000171	uncertain_significance&likely_benign
594F	PTEN-mut	17:78857246A>G	*RPTOR*	0.49	missense	MODERATE	ENSG00000141564	407	P/L	rs922237067&COSM1579117	1.63E-05	
700F	PTEN-mut	17:78820280C>T	*RPTOR*	0.51	missense	MODERATE	ENSG00000141564	1228	V/M	rs147241989	0.000307	
762F	PTEN-mut	19:40761140T>C	*AKT2*	0.48	missense	MODERATE	ENSG00000105221	71	N/S	rs200272953	0.000219	
794F	PTEN-mut	1:11300579C>T	*MTOR*	0.51	missense	MODERATE	ENSG00000198793	523	D/N	rs376836258&COSM3930270	8.12E-06	
836F	PTEN-mut	1:204438908C>T	*PIK3C2B*	0.49	missense	MODERATE	ENSG00000133056	1029	F/L	rs61763420	0.002197	
981F	PTEN-mut	17:78935270G>A	*RPTOR*	0.49	missense	MODERATE	ENSG00000141564	538	I/V			
981F	PTEN-mut	19:1226587C>G	*STK11*	0.50	missense	MODERATE	ENSG00000118046	415	R/G	rs864622448		uncertain_significance
981F	PTEN-mut	17:17131357C>T	*FLCN*	0.49	missense	MODERATE	ENSG00000154803	32	G/E	rs587778366	1.22E-05	
1065F	PTEN-wt	5:38962438T>C	*RICTOR*	0.50	missense	MODERATE	ENSG00000164327	565	Y/C	rs146754529	0.000707	
1172F	PTEN-wt	9:135779171C>T	*TSC1*	0.67	missense	MODERATE	ENSG00000165699	1041	S/R	rs753374839	5.31E-05	uncertain_significance&likely_benign
1173F	PTEN-wt	17:29663905T>G	*NF1*	0.50	missense	MODERATE	ENSG00000196712	2113	C/G	COSM3958176&COSM3958177		
1174F	PTEN-wt	17:17127234A>T	*FLCN*	0.49	splice_donor_variant	HIGH	ENSG00000154803			CS101080		
1197F	PTEN-wt	1:204429727C>T	*PIK3C2B*	0.52	missense	MODERATE	ENSG00000133056	750	G/S	rs114917235	0.000557	
1198F	PTEN-wt	5:38953625G>A	*RICTOR*	0.50	missense	MODERATE	ENSG00000164327	910	R/C	rs143469898	0.000187	
1210F	PTEN-wt	1:204411723G>T	*PIK3C2B*	0.52	missense	MODERATE	ENSG00000133056	458	R/Q	rs61755372	0.000658	
1210F	PTEN-wt	1:204429727C>T	*PIK3C2B*	0.48	missense	MODERATE	ENSG00000133056	8	G/E	rs115204119	0.001045	
1708C	PTEN-wt	14:105240256C>T	*AKT1*	0.49	missense	MODERATE	ENSG00000142208	46	D/E	rs146875699	0.000317	uncertain_significance&not_provided
181F	PTEN-wt	17:29553639A>T	*NF1*	0.48	missense	MODERATE	ENSG00000196712	730	N/Y	rs758893131	0.000061	uncertain_significance
308F	PTEN-wt	5:67522555A>G	*PIK3R1*	0.51	missense	MODERATE	ENSG00000145675	18	R/G			
308F	PTEN-wt	16:2135023A>G	*TSC2*	0.48	missense	MODERATE	ENSG00000103197	1522	N/S	rs144062721&COSM3787107&COSM3787108	6.51E-05	uncertain_significance&not_provided, prob.benign
612F	PTEN-wt	17:17118598C>T	*FLCN*	0.50	missense	MODERATE	ENSG00000154803	445	A/T	rs41419545	0.002648	benign&likely benign&VUS
632F	PTEN-wt	16:2110684C>T	*TSC2*	0.48	missense	MODERATE	ENSG00000103197	330	P/L	rs140910086	3.66E-05	uncertain_significance
672F	PTEN-wt	1:204438340T>A	*PIK3C2B*	0.49	missense	MODERATE	ENSG00000133056	197	Q/H	rs17847778	0.000599	
681F	PTEN-wt	19:1223163C>T	*STK11*	0.49	missense	MODERATE	ENSG00000118046	367	T/M	rs587782835&COSM21358	1.67E-05	uncertain_significance
860F	PTEN-wt	22:32234798C>A	*DEPDC5*	0.49	missense	MODERATE	ENSG00000100150	819	P/T			
995F	PTEN-wt	5:67591042GGAA>G	*PIK3R1*	0.49	inframe_deletion	MODERATE	ENSG00000145675	546	E/-			

Table S9. Germline *PTEN* status and NGS findings in the indicated tumor samples (each one from a different proband).

**Cancer tissue**	**Germline *PTEN* status**	***PTEN* status in the tumor (NGS)** (1)	**Other somatic variants found by NGS**
Thyroid	c.634+5G>A	c.634+5G>A	*BRAF* p.(V600E)
Rectum	WT	WT	*TP53* p.(R282W)
Lung	WT	c.635-1G>A	*ATRX* p.(Q1877H)<br>*PIK3C2B* p.(Q197H)
Thyroid (papillary-follicular)	Large deletion (*KLLN* and *PTEN* exon 1)	*	*PIK3CA* p.(G1049R)<br>*USP9X* p.(I2120fs)

*Large deletions are not called in the NGS pipeline.

(1) In heterozygous status
