# Supplementary material for: Considerations on diagnosis and surveillance measures of PTEN hamartoma tumor syndrome: clinical and genetic study in a series of Spanish patients
Source: Orphanet J Rare Dis. 2022 Feb 28;17:85. doi: 10.1186/s13023-021-02079-7 (PMC8886852; doi:10.1186/s13023-021-02079-7)
Supplement: Supplementary file 2 — Additional file 2: Appendix S1. List of authors and affiliations of the PHTS working group. [file 13023_2021_2079_MOESM2_ESM.docx]

**S1 Appendix: PHTS working group**

**Authors listed in alphabetical order by surname:**

Miguel Ángel Alonso^13^, Raquel Andrés^14^, Sara Arévalo^15^, María del Mar Arias^13^, Judith Balmaña^16^, Elena Beristain^17^, Ignacio Blanco^18^, Mauro Boronat^19^, Joan Brunet^18^, María Victoria Cózar^20^, Miguel del Campo^16^, Arantza Díaz ^21^, Elisabeth Gabau^22^, María Jesús Barcina^23^, Margarita González^24^, Miriam Guitart^22^, Imma Hernán^25^, Héctor Salvador Hernández^26^, Susana Hernando^27^, Carmen Lacambra^28^, Adriana Lasa^29^, Enrique Lastra^30^, Gemma Llort^22^, María del Rosario Marín^31^, David Marrupe^21^, Francisco Martínez^32^, Víctor Martínez^33^, Loreto Martorell^26^, María Orera^34^, Susana Pedrinaci^35^, Pedro Pérez^36^, Marta Pineda^18^, Ana María Plasencia^37^, Teresa Ramón y Cajal^29^, Luis Robles^38^, Diana Rodà^22^, Nuria Rodríguez^33^, Jordi Rosell^39^, Raquel Sáez^15^, Mónica Salvat^40^, Antonio Sánchez^41^, Alfredo Santana^19^, José Luis Soto^42^, Agustín Toll^43^, Anna Tuneu^15^, Carlos Vázquez^19^.

**Affiliations:**

^13^Virgen del Camino Hospital (Pamplona, Spain). ^14^Lozano Blesa Hospital

(Zaragoza, Spain). ^15^Hospital of Donostia (Donostia, Spain). ^16^Vall d’Hebrón Hospital (Barcelona, Spain). ^17^Txagorritxu Hospital (Vitoria-Gasteiz, Spain). ^18^Catalan Institute of Oncology (L’Hospitalet de Llobregat, Spain). ^19^Hospital of Gran Canaria (Las Palmas de Gran Canaria, Spain. ^20^Virgen de Valme Hospital (Sevilla, Spain). ^21^Móstoles Hospital (Móstoles, Spain). ^22^Parc Taulí Hospital (Sabadell, Spain). ^23^Basurto Hospital (Bilbao, Spain). ^24^Can Misses Hospital (Ibiza, Spain). ^25^Terrassa Hospital (Terrassa, Spain). ^26^Sant Joan de Déu Hospital (Esplugues de Llobregat, Spain). ^27^Alcorcón Hospital (Alcorcón, Spain). ^28^Severo Ochoa Hospital (Leganés, Spain). ^29^Sant Pau Hospital (Barcelona, Spain). ^30^Hospital of Burgos (Burgos, Spain). ^31^Puerta del Mar Hospital (Cádiz, Spain). ^32^Nuestra Señora de la Candelaria Hospital (Santa Cruz deTenerife, Spain). ^33^La Paz Hospital (Madrid, Spain). ^34^Gregorio Marañón Hospital (Madrid, Spain). ^35^Virgen de las Nieves Hospital (Granada, Spain). ^36^San Carlos Hospital (Madrid, Spain). ^37^Asturias Central Hospital (Oviedo, Spain). ^38^12 de Octubre

Hospital (Madrid, Spain). ^39^Son Dureta Hospital (Palma, Spain). ^40^Sant Joan de Reus. ^41^Puerta de Hierro Hospital, Madrid, Spain. ^42^General Hospital of Elche (Alicante), Spain. ^43^Del Mar Hospital, Barcelona, Spain.
