# Supplementary material for: Considerations on diagnosis and surveillance measures of PTEN hamartoma tumor syndrome: clinical and genetic study in a series of Spanish patients
Source: Orphanet J Rare Dis. 2022 Feb 28;17:85. doi: 10.1186/s13023-021-02079-7 (PMC8886852; doi:10.1186/s13023-021-02079-7)
Supplement: Supplementary file 3 — Additional file 3: Appendix S2. References related to supporting information. [file 13023_2021_2079_MOESM3_ESM.docx]

**S2 Appendix. References related to supporting information**

1. Tan MH, Mester J, Peterson C, Yang Y, Chen JL, Rybicki LA, et al. A clinical

scoring system for selection of patients for PTEN mutation testing is proposed

on the basis of a prospective study of 3042 probands. Am J Hum Genet.

2011;88(1):42–56.

2. Pilarski R, Burt R, Kohlman W, Pho L, Shannon KM, Swisher E. Cowden

syndrome and the PTEN hamartoma tumor syndrome: systematic review and

revised diagnostic criteria. J Natl Cancer Inst. 2013;105(21):1607–16.
