# Supplementary material for: Considerations on diagnosis and surveillance measures of PTEN hamartoma tumor syndrome: clinical and genetic study in a series of Spanish patients
Source: Orphanet J Rare Dis. 2022 Feb 28;17:85. doi: 10.1186/s13023-021-02079-7 (PMC8886852; doi:10.1186/s13023-021-02079-7)
Supplement: Supplementary file 4 — Additional file 4: Methods S1. Clinical questionnaire. [file 13023_2021_2079_MOESM4_ESM.docx]

**S3 Appendix: Clinical questionnaire**

The original questionnaire distributed to the physicians was a document of 3 pages in Spanish, formatted and edited with tables and enough blank space for the answers.

Herein we enumerate in English the same information requested.

Patient ID

Birth date

Sex

Date of clinical examination

Name of physician

Medical centre

Answer “yes” for presence or “no” for absence, and indicate “number, body localization, date/age at diagnosis, histologic type and any other relevant information”, for the following features:

Skin and mucosa:

Facial papules

Oral papillomas

Acral keratoses

Trichilemmomas

Macular pigmentation of penis

Lipomas

Fibromas

Neuromas

Others

Genitourinary system:

Ovarian cysts

Uterine fibromas/miomas

Testicular lipomatosis

Others

Thyroid:

Nodules

Multinodular goiter

Adenomas

Hashimoto’s thyroiditis

Others

Digestive tract:

Polyps

Esophageal glycogenic acanthosis

Others

Lipovascular lesions:

Lipomatosis

Vascular malformations

Others

Neurological disorders:

Lhermitte-Duclos

Mental retardation

Psicomotor retardation

Autism

Epilepsy

Others

Fibrocystic breasts

Macrocephaly

Cephalic perimeter at birth

Cephalic perimeter at date of exploration

General body overgrowth

Height and weight at birth

Height and weight at date of exploration

Cancer:

Breast

Endometrial

Ovarian

Thyroid

Colorectal

Renal

Melanoma

Others
