# Supplementary material for: Considerations on diagnosis and surveillance measures of PTEN hamartoma tumor syndrome: clinical and genetic study in a series of Spanish patients
Source: Orphanet J Rare Dis. 2022 Feb 28;17:85. doi: 10.1186/s13023-021-02079-7 (PMC8886852; doi:10.1186/s13023-021-02079-7)
Supplement: Supplementary file 5 — Additional file 5: Methods S2. Primers and further information regarding the NGS and WES. [file 13023_2021_2079_MOESM5_ESM.docx]

**S2 Methods. Primers and further information regarding the NGS and WES**

**Next generation sequencing (NGS) panel**

In order to obtain good yields for NGS, DNA samples were prepared as follows.

Whenever the samples were eluted in EDTA and corresponding blood samples were available, DNA was re-extracted using the Maxwell® 16 Blood DNA Purification Kit in combination with the Maxwell® 16 Instrument (Promega) and eluted in 0.1 M Tris-HCl pH=8.0. DNA samples referred from outside our laboratory, with no knowledge of their elution buffer, were purified using a ratio of 1.6X Promega magnetic beads. Quant-iT PicoGreen dsDNA reagent (Thermo Fisher) was used to quantify the dsDNA concentration for each sample. The library was prepared following the SeqCap EZ HyperCap Workflow User’s Guide (Roche), using 250 ng of input germline genomic DNA.

Library sequencing was performed in a HiSeq 2500 Illumina Sequencing Instrument with a resultant coverage of up to 900X for blood samples and 700X for tumor samples, 4 million paired-end reads and 15% of duplicates approximately for each sample. Another quality control was performed using FASTQC software to check correct sequencing of every sample (coverage, duplicates, phred, etc.). The Integrative Genomics Viewer (IGV; Broad Institute) was used to visually check the NGS data for possible artifacts (duplicates, repetitive regions, low read depth or poor coverage). Germline variant annotation was performed separately for SNVs and indels, using the following tools: BWA, SAM, PICARD, GATK, HaplotypeCaller and VEP.

**Whole exome sequencing (WES)**

Sequencing libraries were generated using the Agilent SureSelect Human All Exon kit (Agilent Technologies). The probands’ WES data were grouped as shown in Fig S1 in order to search for shared variants, genes or pathways affected. In the variant filtering, we assumed a monogenic model of the disease and excluded variants that were in non-canonical transcripts or in non-exonic regions; synonymous variants, variants with low read depth or a MAF > 0.001 in the European population (according to gnomAD, ExAC and dbSNP) were also excluded. We prioritized variants with *in silico*-predicted deleteriousness (through SIFT, Polyphen2, Condel, FATHMMM, MutationTaster, and MutationAssessor) and that appeared in genes related with PTEN function or pathway (using STRING, Reactome and Kegg pathway), or in known cancer genes, or that were related with the patient phenotype, such as autism genes (using ClinVar, OMIM and HPO).
